# A genetic risk assessment for prostate cancer influences patients’ risk perception and use of repeat PSA testing: a cross-sectional study in Danish general practice

**DOI:** 10.3399/bjgpopen20X101039

**Published:** 2020-05-27

**Authors:** Jacob Fredsøe, Pia Kirkegaard, Adrian Edwards, Peter Vedsted, Karina Dalsgaard Sørensen, Flemming Bro

**Affiliations:** 1 Department of Molecular Medicine, Aarhus University Hospital, Aarhus, Denmark; 2 Department of Clinical Medicine, Aarhus University, Aarhus, Denmark; 3 Department of Public Health Programs, Randers Regional Hospital, Randers, Denmark; 4 Division of Population Medicine, School of Medicine, Cardiff University, Cardiff, UK; 5 Department of Public Health, Research Centre for Cancer Diagnosis in Primary Care, Aarhus University, Aarhus, Denmark; 6 Department of Public Health, Research Unit for General Practice Aarhus University, Aarhus, Denmark

**Keywords:** prostate-specific antigen, prostatic neoplasms, risk assessment, general practice, surveys and questionnaires, genetic testing

## Abstract

**Background:**

Prostate cancer (PC) is the most common cancer among men in the western world. Genetic lifetime risk assessment could alleviate controversies about prostate specific antigen (PSA) testing for early diagnosis.

**Aim:**

To determine how men interpret information about their lifetime risk for PC and how this can affect their choice of having a repeated PSA test.

**Design & setting:**

A genetic test was offered for assessment of individual PC lifetime risk in general practices in Denmark, with the purpose of promoting appropriate use of PSA testing.

**Method:**

Participants had a genetic lifetime risk assessment for PC diagnosis (either high or normal risk). A month after receiving the result, participants answered a questionnaire about their perceived risk of getting or dying from PC compared with other men, as well as their intentions for repeated PSA testing.

**Results:**

Nearly half (44.7%) of 555 participants who received the genetic risk assessment were not aware they had a genetic test. Nevertheless, compared with men with a normal genetic risk, those with high genetic risk reported higher perceived risk for PC (mean difference of 0.74 [95% confidence interval {CI} = 0.56 to 0.96] on a 5-point scale), higher perceived risk of dying from PC (mean difference of 0.48 [95% CI = 0.29 to 0.66] on a 5-point scale), and increased intention for repeated PSA testing (mean difference of 0.48 [95% CI = 0.30 to 0.65] on a 4-point scale).

**Conclusion:**

Despite low awareness and/or understanding of the test result, a high genetic risk for PC made participants more aware of their risk, and it increased their intention and probability for repeated PSA testing.

## How this fits in

Most guidelines advise against population-based screening using the PSA test. Here, a genetic test was offered for assessment of individual PC lifetime risk in Danish general practice, with the purpose of promoting appropriate use of PSA testing. A month after receiving the result, participants answered a questionnaire about their perceived risk of getting or dying from PC, as well as their intentions for repeated PSA testing. Despite low awareness and/or understanding of the test result, having a high genetic risk for PC made participants more aware of their risk, and it increased their intention and probability for repeated PSA testing.

## Introduction

PC is the most common cancer among men in Europe, with approximately 190 000 new cases and about 80 000 deaths every year.^1–4^ A commonly used method for early detection of PC is the PSA test, although this method has limited accuracy.^5,6^ This results in failure to detect genuinely aggressive disease at an early stage, as well as overdiagnosis and overtreatment of indolent cancers that would not give rise to symptoms in the patient’s normal lifespan if left undetected. Therefore, most guidelines advise against population-based screening using the PSA test.^7,8^ Still, the PSA test is frequently requested by some men and opportunistically offered by GPs, typically in relation to lower urinary tract symptoms (LUTS) or regular health checks.^9^

Risk stratification has been proposed as a strategy to improve the benefit-to-harm ratio of (opportunistic) PSA screening by targeting PSA testing to those men most likely to benefit.^10^ Genetic markers are candidates to provide such risk assessment for PC, and advances in genome-wide association studies have so far identified around 200 genetic variants associated with higher PC lifetime risk, termed single nucleotide polymorphisms (SNPs).^11–14^ Furthermore, retrospective studies comparing non-genetic risk prediction models versus risk prediction models, including genetic markers (SNPs), have reported significantly higher specificity for the genetic models.^15–17^ From a patient perspective, knowledge of one’s lifetime PC risk could enhance accuracy of perceived susceptibility^18,19^ and aid in shared decision-making about PSA testing.^20^

To the authors' knowledge, no previous clinical studies have investigated if a genetic PC risk assessment can be utilised as a tool for optimising the use of PSA testing in a general practice setting. An intervention was previously developed^21,22^ in which a genetic lifetime risk assessment for PC was offered to patients who had a PSA test at their general practice. The aim was to enhance appropriate PSA testing by: (a) reducing the number of unnecessary PSA tests (opportunistic screening) in men with a normal lifetime risk; and (b) promoting targeted PSA screening of high-risk men (≥30% lifetime risk) to ensure early detection. The result of a genetic PC risk assessment could then support decisions where both patient and GP had additional information about the future risk of getting a PC diagnosis.

The aim of this study was to explore whether patients were aware of the genetic test, and how information about lifetime risk influenced their perceived risk of PC and their intentions to have a repeated PSA test in the future.

## Method

### Study setting and participants

The study was performed in 73 general practices in the Central Region of Denmark. Citizens are registered with a specific general practice, which they have to consult for medical advice, and the GP acts as gatekeeper for the rest of the healthcare system.

All patients were included from the intervention arm of the ProCaRis project^21^ from January 2013 to December 2013. The aim of the ProCaRis project was to identify a group of patients for whom PSA testing would have most benefit, by introducing a genetic test for lifetime risk of PC in general practice. In brief, all patients who received a PSA test at their general practice within the study period were eligible for inclusion, unless they showed one of the following exclusion criteria: aged >80 years; elevated PSA level (≥4 µg/l) concurrently or within the previous 2 years; known prostate or bladder disease; or a history of PC. As little control as possible was asserted over the entire inclusion process to minimise any disturbances to the normal workflow and, consequently, the reasoning behind the initial PSA test was not known to the authors; it was left solely to the GP and patient’s discretion. In addition to having a PSA test, eligible patients had an additional 4 ml blood sample drawn for a genetic risk assessment.

Based on genotyping of 33 risk loci and/or SNPs (adjusted for age and PC family history), the individual lifetime PC risk was calculated for each participant as previously described,^21,23^ and results reported as high, normal, or unknown risk. As the lifetime risk score was adjusted for age in the algorithm, no further distinction was made between younger and older participants. A high lifetime risk was considered as an absolute lifetime risk of ≥30%, which is nearly three times higher than the average lifetime risk of 11% and thus comparable with the Danish recommendation to only offer PSA testing in men aged >45 years, with a family history of PC (two close relatives), and to those with a life expectancy of >10–15 years.^8,24–26^ A lifetime risk <30% was considered 'normal'. In the rare cases, where the patient’s family disposition was unknown but the total risk assessment, in theory, would change from ‘normal’ to ‘high’ in the case of a known positive family history, the total risk assessment result was reported as ‘unknown’. Accordingly, after determining the risk profile (with a mean response time of 21 days after the blood sampling), one of the following messages was sent electronically to the GP:

#### Normal lifetime risk

*'The patient belongs to a group of patients at normal lifetime risk of getting a prostate cancer diagnosis. It is not considered necessary or beneficial for the patient to have more PSA tests in the future, unless the patient develops urinary tract symptoms or one or more of his relatives develops prostate cancer*.'

#### High lifetime risk

*'The patient belongs to a group with increased risk of developing prostate cancer in the future. If the patient develops prostate cancer in the future, in most cases, the cancer will be slow growing. For early detection, the patient is encouraged to have a yearly PSA test.'*

#### Unknown lifetime risk

*'The risk for developing prostate cancer in the future cannot be estimated due to missing information for family history.*'

The GP or their staff then informed the patient about the result by telephone, email, letter, or during consultation. Before the study, each practice had received written information with recommendations about follow-up PSA testing, according to current guidelines and information about the benefits and shortcomings of genetic risk assessment as a tool to support decision making about testing for PC. The researchers provided additional online information about PC screening, use of PSA tests, and genetic risk assessment for PC, as well as a project telephone hotline.^21,27^

### Measures

PSA test results were collected from the regional clinical laboratory information system (LABKA) and included the PSA level at the time of inclusion and any PSA test(s) performed during the following 2 years after the patient’s initial PSA test.

Questionnaires were sent to participants about 1 month after the genetic test result was available to the GP (and thus, approximately 2 months after initial inclusion); a reminder was sent 2 weeks later if the questionnaire had not been returned. The questionnaires contained questions about patients’ awareness of their lifetime risk of PC, their perceived risk of developing PC, of their overall health, and of their intentions to have a repeat PSA test in the future (Table 1).

**Table 1.** Summary of patient questionnaire used in this study. The full questionnaire participants received is available on request.

**Question label**	**Text**	**Response**
q01	Did you have a PSA test?	1 = yes, 0 = no
q02	Why did you take the PSA test?	
*q02_1*	I have / had urinary problems	
*q02_2*	I got the test, as I still was at the doctor in connection with another health problem	
*q02_3*	I got it taken in conjunction with a regular health check	
*q02_4*	I got the test because I was worried that I have PC	
*q02_5*	I got it taken to be sure that I do not have PC	
*q02_6*	I had not even considered getting test before my doctor recommended me	
*q02_7*	My family has advised me to take the test	
*q02_8*	My friends / acquaintances have advised me to take the test	
*q02_9*	I have friends / acquaintances who have had PC and are living with it today	
*q02_10*	I have friends / acquaintances who have died of PC	
*q02_11*	I have had PC	
*q02_12*	I have had another type of cancer	
*q02_13*	I have had an elevated PSA level	
*q02_14*	Other reason. Please specify	
q03	How were the results of the PSA test?	1 = normal, 2 = increased, 9 = don't know
q04	How do you assess your risk of getting PC, compared with other men your age?	1 = much lower, 2 = lower, 3 = the same, 4 = higher, 5 = much higher
q05	How do you assess your risk of dying from PC compared with other men your age?	1 = much lower, 2 = lower, 3 = the same, 4 = higher, 5 = much higher
q06	Although my PSA level is normal now, I am determined to ask my doctor for a new PSA test over the next 2 years	1 = totally disagree, 2 = disagree, 3 = agree, 4 = totally agree, 9 = don't know
q07	How do you think your health is overall?	1 = bad, 2 = less good, 3 = good, 4 = very good, 5 = excellent
q08	Have you had taken a genetic test?	1 = yes, 0 = no
q09	How was the result of your genetic testing?	1 = normal, 2 = increased, 9 = don't know

PC = prostate cancer. PSA = prostate specific antigen.

The genetic lifetime risk (normal, high, or unknown) results were collected directly from the laboratory at the Department of Molecular Medicine, Aarhus University Hospital, Denmark, where all genetic analyses were performed.

### Statistical analysis

All statistical analyses were performed in R^28^ (version 3.5.1) using R studio (version 1.1.383). Fisher's exact test was used to test differences in distributions, while differences in means or medians were tested by Student *t*-test or Wilcoxon rank sum test, respectively. CIs were calculated from a two-sided Student *t*-test.

Univariate and multivariate Cox regression analyses were performed using the survival package,^29^ and the endpoint was a repeat PSA test within 2 years. Patients who did not have a repeat PSA test within that timeframe were censored at 730 days (that is, 2 years). *P* values <0.05 and/or differences outside 95% CIs were considered significant.

To explore which factors were associated with patients’ decisions to have a repeat PSA test, a multivariate Cox regression model was created using a repeat PSA test within 2 years as endpoint. Consequently, the study used genetic risk, perceived risk for PC diagnosis, perceived risk for dying of PC, intention for a repeat PSA test, PSA level at inclusion, and awareness of having had a genetic test as possible explanatory variables.

## Results

### Questionnaire response rate

**Figure 1.**
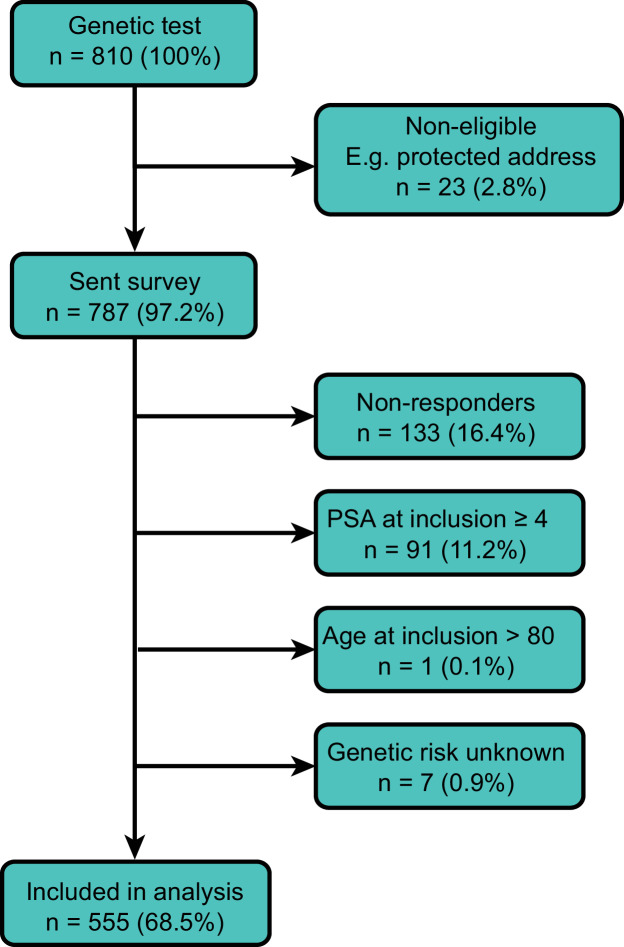
Flow chart of inclusion and exclusion criteria. PSA = prostate specific antigen.

In total, 810 patients received a genetic test in 73 general practices. Of these, 787 patients (97.2%, Figure 1) were eligible and received the questionnaire (Table 1). After removing non-responders (*n* = 133, 16.4%), patients with PSA ≥4 µg/l at inclusion (*n* = 91, 11.2%), patients aged 80 years (*n* = 1, 0.1%), or unknown genetic risk (*n* = 7, 0.9%), a total of 555 patients (68.5%) were included in the analysis (Table 2).

**Table 2.** Summary of patient characteristics (*N* = 555)

****Characteristics	*n* (%)**^a^**
**Median a** **ge at inclusion,** **years** **(** **IQR** **)**	63.4 (56.7 to 68.9)
**Median** **PSA at inclusion** **(** **IQR** **)**	1.1 (0.7 to 2.0)
**PSA levels**	
<1 ng/ml	213 (38.4)
≥1 ng/ml	342 (61.6)
Unknown	0 (0.0)
**Lifetime PC** **risk**	
Average risk	482 (86.8)
High risk	73 (13.2)
Unknown	0 (0)
General wellbeing (scale 1–5), mean (SD)	3.39 (0.79)
**Reason to get PSA test** ^b^	
Urinary problems	185 (33.3)
Saw the doctor for another health problem	140 (25.2)
As part of a regular health check	193 (34.8)
Worried about or ruling out having PC	351 (63.2)
Not intended before doctor recommendation	102 (18.4)
Family or friends or acquaintances advise	142 (25.6)
Friends or acquaintances living with or died from PC	245 (44.1)
Having another type of cancer	7 (1.3)
Previous elevated PSA level	8 (1.4)
Other reasons	60 (10.8)

^a^Unless stated otherwise. ^b^Participants able to select multiple reasons. IQR = interquartile range. PC = prostate cancer. PSA = prostate-specific antigen. SD = standard deviation.

### Patients’ understanding of their test results

**Figure 2.**
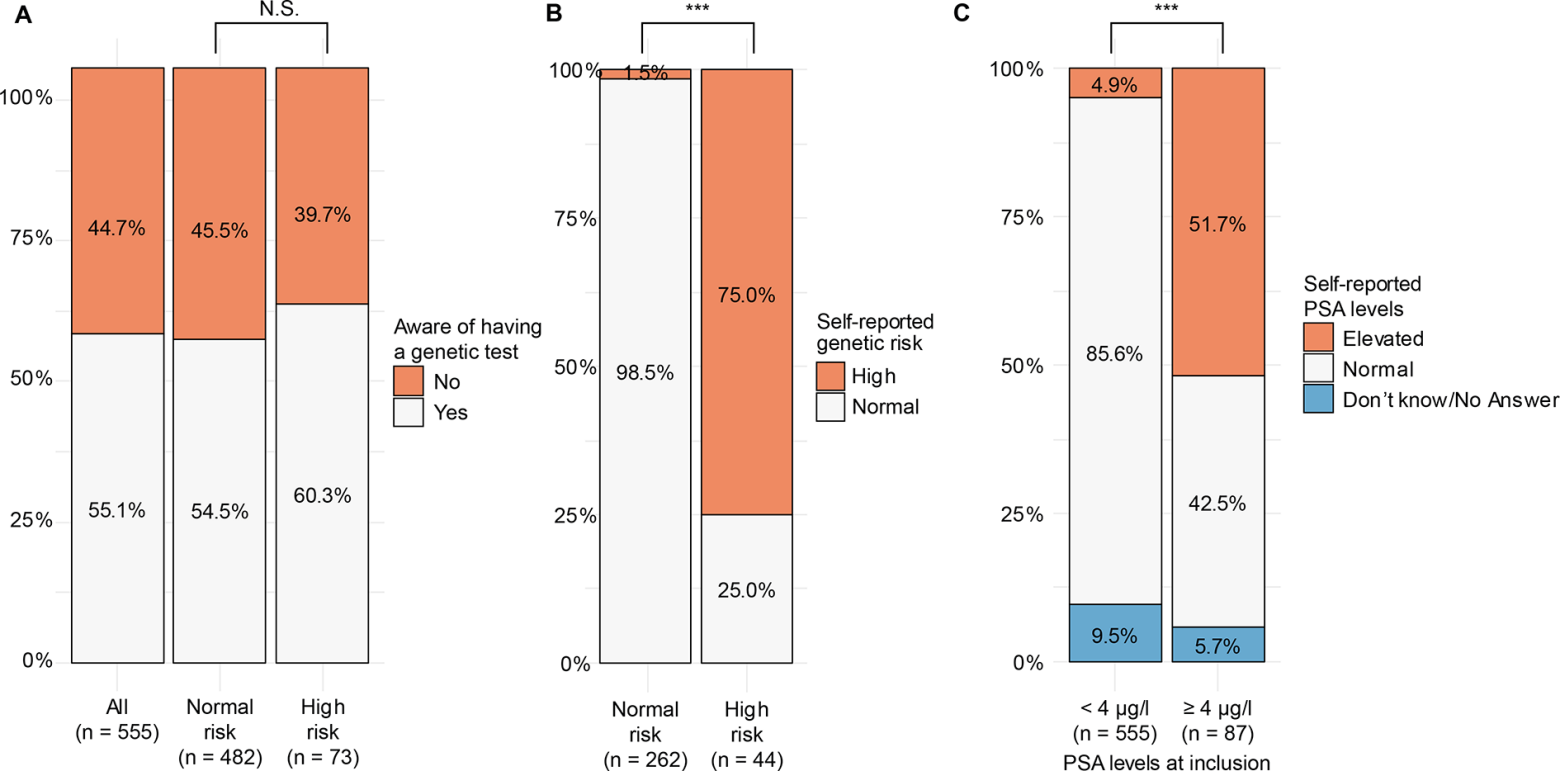
Self-reported outcome of tests for PC. In total, 555 patients (482 with a normal risk, 73 with a high risk) were asked if they had received a genetic test (A). Patients who were aware of the genetic test (262 normal risk and 44 high risk) were next asked about the result of the test (B). Out of all questionnaire responders, patients reported if their PSA levels were normal, elevated, or they did not know (or did not answer) (C). Answers were separated into bins, based on PSA level at inclusion. Fisher’s exact test was used to test for difference in distribution. NS = not significant. PC = prostate cancer. PSA = prostate specific antigen. ***P<0.001.

Slightly more than half of the responders (*n* = 306, 55.1%) recalled they had a genetic test performed (q08, Table 1 and Figure 2a). The awareness of having had a genetic test was not significantly different (Fisher’s test, *P* = 0.379) for patients with a high versus a normal risk test result (Figure 2a). When the patients who were aware they had received a genetic test were asked about the result of their genetic test (that is, lifetime genetic risk), patients with a normal genetic lifetime risk correctly reported their risk in 98.5% (*n* = 258/262) of cases, while a significantly smaller fraction (75.0%, *n* = 33/44) of patients with high genetic risk reported correctly (Figure 2b; *Δ* = 0.23; *t*-test, *P*<0.001; 95% CI = 0.10 to 0.37).

For comparison, the patients’ knowledge about their PSA test result at inclusion was also analysed. For this analysis only, all responders were incorporated, including also those (*n* = 87) with a PSA level ≥4 µg/l at inclusion. Only 51.7% of patients with PSA levels ≥4 µg/l correctly reported their PSA levels as elevated (Figure 2c). In contrast, participants with normal PSA levels (<4 µg/l) correctly reported their PSA levels as normal in 85.6% of the cases (Figure 2c; *change (Δ)* = 0.34; *t*-test, *P*<0.001; 95% CI = 0.23 to 0.45).

### The patients’ perceived risk of PC correlated with actual genetic risk

**Figure 3.**
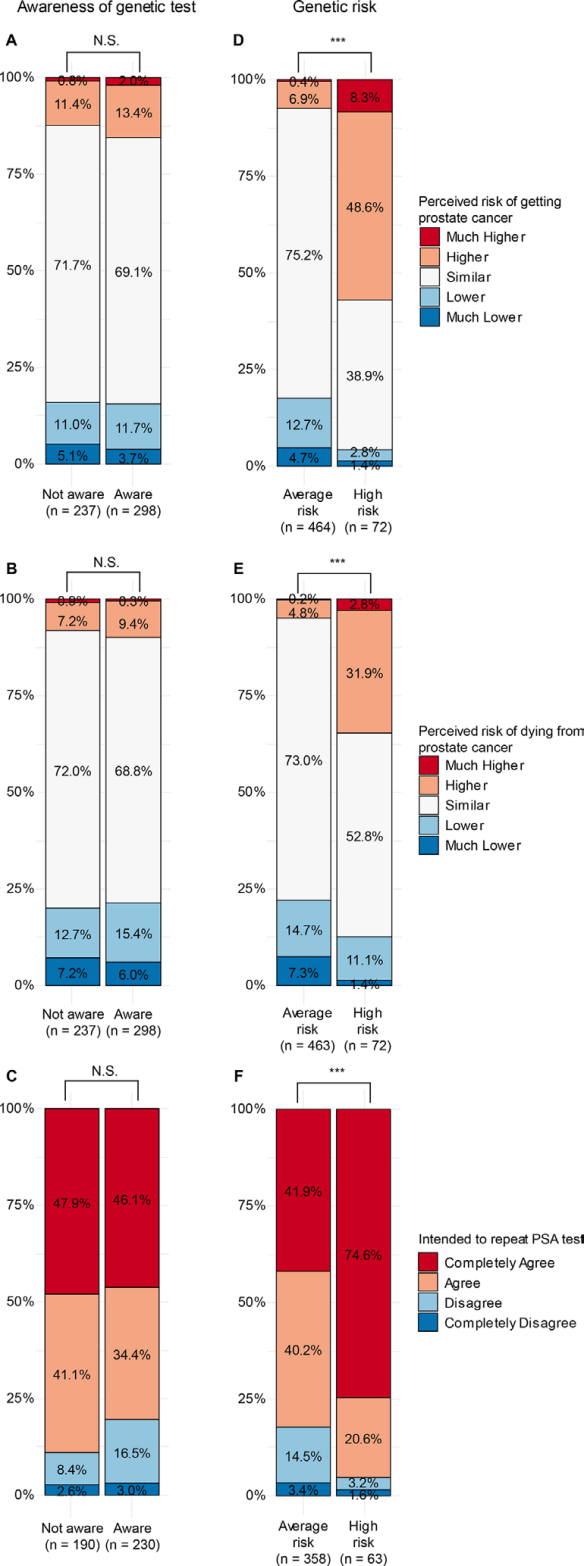
Self-reported perceived risk of getting a PC diagnosis, dying of PC, or intention of a repeat PSA test. Patients were asked about their perceived risk of getting PC and/or dying from PC on a scale of 1–5, as well as their intent to have a repeat PSA test within 2 years on a scale of 1–4. The perceived risks and intention were plotted against awareness of having the genetic test (A-C), or the actual test result of measured genetic risk (D-F). Fisher’s exact test was used to test for difference in distribution. PC = prostate cancer. PSA = prostate specific antigen. NS = not significant. ***P<0.001.

No significant association was found between whether the patients were aware they had received a genetic test and their perceived risk of being diagnosed with PC (Figure 3a, *P* = 0.68; Fisher’s exact test), or dying of PC (Figure 3b, *P* = 0.65), nor with their intention to have a repeat PSA test within 2 years (Figure 3c, *P* = 0.08). In contrast, a strong positive correlation was found between the patients’ actual measured genetic risk and their perceived risk of getting PC (Figure 3d, *P* = 2.6E-20; Fisher’s exact test) and dying of PC (Figure 3e, *P* = 3.9E-9), as well as their intention for a repeat PSA test (Figure 3f, *P* = 2.2E-5). A possibility remains that this association was driven by the group of patients who were aware they had a genetic test; however, when the patients were stratified based on awareness, the correlation between the patients’ actual measured genetic risk and their perceived risks of getting PC and dying of PC remained significant regardless of awareness (*P*≤0.01 in all cases). In contrast, the intention to repeat the PSA test was only significantly associated with genetic risk in the subgroup of patients who were aware that they had had a genetic test (*P* = 5.3E-5).

In a study of the same cohort by a number of the present authors,^23^ a strong association between the genetic risk and whether a patient had a repeat PSA test was observed. Accordingly, 83.6% (61 out of 73) of patients with a high genetic risk had a repeat PSA test within 2 years, while 20.5% (99 out of 487) of patients with a normal risk had a repeat PSA test (*Δ* = 0.63; *t*-test, *P*<0.001; 95% CI = 0.54 to 0.72).

### Regression model for repeat PSA test

A multivariate Cox regression model revealed that a high genetic risk score was the most significant predictor of a repeated PSA test (hazard ratio (HR) = 5.99; 95% CI = 4.09 to 8.79, *P*<0.001, Table 3). In addition, the intention for a repeat PSA test was also a significant independent predictor for having a repeat PSA test (HR = 1.2; 95% CI = 1.05 to 1.37, *P* = 0.007, Table 3). Awareness of having the genetic test and PSA level at inclusion (HR = 1.16) were borderline significant predictors of a repeat PSA test (*P*<0.1) in multivariate analysis, whereas perceived risk of getting PC or dying from PC were non-significant (*P*≥0.1). For those who had a repeat PSA test, the median time to repeat PSA was 380 days (IQR 338 to 505 days) after the genetic risk assessment.

**Table 3.** Cox regression analysis for repeat PSA test (*N* = 555, 160 with repeat PSA test). Uni- and multi-variate Cox regression, using genetic risk score, awareness of having a genetic test, PSA level at inclusion, perceived risk of PC, perceived risk of dying from PC, and intention for a repeat PSA were investigated as potential explanatory variables for actually having a repeat PSA test within 2 years.

	**Univariate**	**Multivariate**
**Variable**	**Characteristics**	**HR (95% CI**)	***P* value**	**C-index** ^a^	**HR (95% CI**)	***P* value**
Genetic risk	Normal versus high	8.11(5.83 to 11.29)	**<** **0.001**	0.66	5.99(4.09 to 8.79)	**<** **0.001**
Awareness of having a genetic test	No versus yes	1.23(0.90 to 1.69)	0.193	0.53	1.32(0.95 to 1.83)	0.099
PSA at inclusion	Continuous	1.24(1.05 to 1.46)	**0.0097**	0.56	1.16(0.98 to 1.38)	0.083
Perceived risk for PC	1–5	2.13(1.68 to 2.71)	**<** **0.001**	0.62	1.2(0.83 to 1.74)	0.331
Perceived risk for dying of PC	1–5	1.77(1.38 to 2.28)	**<** **0.001**	0.59	1.11(0.79 to 1.57)	0.553
Intention for repeat PSA test	1–4	1.35(1.18 to 1.54)	**<** **0.001**	0.62	1.2(1.05 to 1.37)	**0.007**

^a^C-index, Harrell’s concordance index. HR = hazard ratio. PC = prostate cancer. PSA = prostate specific antigen.

## Discussion

### Summary

This study found that almost half of the participants were unaware they had a genetic assessment of their lifetime risk for PC. Of those who were aware, up to 25% did not correctly report their risk; however, the results showed that the actual genetic risk, and not an awareness of having the genetic test correlated with the patients’ perceived risk of PC, their intention to repeat their PSA test, and whether they then had a repeat PSA test. Together, this appears to indicate that patients followed the advice given by their GP (that is, to repeat the PSA test or not), despite not necessarily comprehending the test result. Alternatively, the GPs may not have found it essential to convey the details of the test result and simply gave patients the recommendations.

### Strengths and limitations

To the best of the authors' knowledge, this is the first prospective study using a genetic risk test to promote targeted PSA screening of high-risk individuals in general practice, coupled with assessment of patient behaviour. A strength of this study is the availability of data from registries in addition to the questionnaires. This enhanced the validity of the data, especially because many patients were not aware of the genetic test result. Furthermore, using registries made it possible to collect data at different time points (the genetic test and data on any follow-up PSA test 2 years later).

Since the inception of this study, more PC-related SNPs have been identified,^12–14^ making risk assessment potentially more precise. However, as the endpoint in the present study was patient behaviour and not PC diagnosis, the accuracy of the risk assessment is not expected to influence the results presented here.

The risk of performance bias was low as the intervention comprised only paper-based information and clinical advice, and no education or training of GPs was required. However, this will often be the case when new tests are introduced in health services.

### Comparison with existing literature

The low awareness of participant’s genetic test result is consistent with a previous study where patients had poor recall of risk figures given to them in genetic counselling sessions.^30^ This raises questions over whether patients actually understand the meaning and purpose of the test. A potential reason for this low awareness could be that the decision to test was not based on patients’ requests; the tests were offered by their GP. The authors speculate that patients might have taken the test simply because more testing is felt to be 'better'.^22,31^

The strong direct effect of lifetime risk for PC on follow-up PSA tests, despite the patients’ limited awareness of having the genetic test, suggests that GPs play an important role in the decision about follow-up PSA testing. While it is not known how (and whether) the GPs conveyed the purpose of the test and the result to the patients, patient behaviour and risk perception was changed following the genetic risk assessment process. This likely indicates that patients largely followed their GPs' recommendations. This is even more evident for patients who were not aware of their normal (that is, non-elevated) genetic risk, but still had fewer PSA tests within 2 years.^23^ The relatively smaller indirect effect through perceived risk is consistent with earlier studies that found limited effects of long-term genetic testing on perceived risk.^32^

### Implications for research and practice

The simplicity of the intervention, combined with the differences between patients with normal and high lifetime risk at follow-up PSA testing, makes this intervention a candidate for broader implementation to enhance appropriate PSA testing. However, before doing this, further research about the potential effects of this genetic test is required. First, the low awareness among patients receiving a genetic test provides a challenge for broader implementation of this test. It is possible that the GP did not use the wording 'genetic test' per se, but rather a lifetime risk test and, as such, it is not expected from the patients to know they had a genetic test. However, it is believed that the implications of the low awareness of a genetic test would have little impact on the decision-making process. Rather, a tangible recommendation is provided (that is, have an annual PSA test, or no benefit from screening). The effect of this recommendation can be seen in the data.

Further studies are required to determine how to communicate the test results effectively to patients, and to evaluate whether the genetic test could be integrated with a decision aid to support informed decision-making between men and their GP about appropriate PSA testing. Second, this report did not focus on the effect of the intervention on overall PSA test usage, as this has already been addressed elsewhere.^23^ Future studies should assess both the effects of communication strategies and offering a genetic test for lifetime PC risk on the number of PC diagnoses. Ideally, such future studies should include an optimised genetic test that includes all currently known PC risk variants.

Despite limited understanding of test results, offering a genetic test to assess the lifetime risk of PC altered the behaviour of patients about having a repeat PSA test within 2 years. Such an intervention could, in the future, form the basis for actively supporting informed decision-making between men and their GP in routine practice.
